# *Lutzomyia longipalpis* (Diptera: Psychodidae) Argentina-Bolivia border: new report and genetic diversity

**DOI:** 10.1590/0074-02760190184

**Published:** 2019-09-30

**Authors:** María Gabriela Quintana, Angélica Pech-May, Ana Denise Fuenzalida, José Manuel Direni Mancini, Paola Andrea Barroso, Zaida Estela Yadón, Mario Zaidenberg, Oscar Daniel Salomón

**Affiliations:** 1 Instituto Nacional de Medicina Tropical, Puerto Iguazú, Misiones, Argentina; 2 Universidad Nacional de Tucumán, Instituto Superior de Entomología, San Miguel de Tucumán, Tucumán, Argentina; 3 CONICET, Buenos Aires, Argentina; 4 Instituto de Patología Experimental, Salta, Salta, Argentina; 5 Pan American Health Organization, Communicable Diseases and Environmental Determinants of Health Department, Washington, DC, USA; 6 Ministerio de Salud y Acción Social, Buenos Aires, Argentina; 7 Red de Investigación de la Leishmaniasis en la Argentina

**Keywords:** visceral leishmaniasis, spatial distribution, Salta

## Abstract

American visceral leishmaniasis (AVL) has two main scenarios of transmission as follows: scattered cases in rural areas and urban outbreaks. Urban AVL is in active dispersion from the northeastern border of Argentina-Paraguay-Brazil to the South. The presence of *Lutzomyia longipalpis* was initially reported in urban environments in the northwestern border of the country. The presence of *Lu. longipalpis*, environmental variables associated with its distribution, and its genetic diversity were assessed in Salvador Mazza, Argentina, on the border with Bolivia. The genetic analysis showed high haplotype diversity, low nucleotide diversity, and low nucleotide polymorphism index. We discuss the hypothesis of an expanding urban population with introgressive hybridisation of older haplogroups found in their path in natural forest or rural environments, acquiring a new adaptability to urban environments, and the possibility of changes in vector capacity.

American visceral leishmaniasis (AVL) due to *Leishmania infantum* is mainly transmitted by the species complex, *Lutzomyia longipalpis* (Lutz & Neiva). AVL has two main scenarios of transmission as follows: scattered human and canine cases in rural areas and urban outbreaks with high prevalence in dogs. The former scenario has been reported since the beginning of the twentieth century, but the latter has been recorded since the 1970s in the northeast Brazil when the urban dispersion of visceral leishmaniasis (VL) in Paraguay, Argentina, and Uruguay began at the turn of the century.^1^

The northern Argentina border extends for 3,573 km and is shared with Bolivia (Northwest), Paraguay (North Central and Northeast), and Brazil (Northeast). Seven human VL cases associated with rural transmission scenarios were recorded in northwest Argentina close to the border with Bolivia between 1923 and 1932.^2^ In contrast, regarding urban transmission in Argentina, the presence of *Lu. longipalpis* was reported in 2004 in the North Central region, in a city on the border with Paraguay, and the first urban human VL case was diagnosed in 2006 in a city located on the northeast border with Paraguay. Since then, the dispersion of urban *Lu. longipalpis*, and human and canine VL cases have continued in the eastern border of the country at a 38-39º south latitude.^3^ However, between 2008-2011, on the northwest Argentina border, three rural human VL cases were reported again after 80 years, remarkably close to Bolivia, without any captures of *Lu. longipalpis*.^4^ ^,^ ^5^ It was transmitted 700 km from the eastern foci related to the urban dispersion of the vector. Further, in 2013, urban *Lu. longipalpis* was initially reported in the Northwest region, in the city of Tartagal, located 55 km from Bolivia and 80-150 km from the rural cases noted in 2008-2011.^6^ Specimens of *Lu. longipalpis* from Tartagal showed higher haplotype diversity (*Hd*) than those from the urban eastern ‘spreading wave’, as if the population of Tartagal belonged to a long-established population, but also with an excess of low-frequency haplotypes as a population of relatively recent expansion.^7^

To explain the above contradiction and consider the geographical distribution of haplotypes, we postulated the hypothesis that the populations of *Lu. longipalpis* that earlier adapted to the rural environment in the northwestern Argentina-Bolivia border could have received newer inputs from the later invasive expansion of urban populations. Therefore, to evaluate this hypothesis, a phlebotomine survey was conducted in Salvador Mazza, Argentina, on the border with Bolivia, to assess the presence of *Lu. longipalpis*, its spatial distribution, associated environmental variables, and its genetic diversity.

Phlebotomine captures were performed on April 2016 in Salvador Mazza, Salta, Argentina, located < 100 m from the border with Bolivia. The study area is in the transitional environment between the Yungas tropical forest and xerophilic Dry Chaco ecoregions. Sixteen sites were randomly selected by visual interpretation within the city and surroundings representative of urban, periurban, and mixed landscapes (Fig. 1).

One ‘REDILA-BL’ trap^8^ was placed on each site for three consecutive nights. The captured phlebotomines were dissected, and the head and last three abdominal segments were clarified with lactophenol and mounted on slides for the identification at the species level,^9^ while the thorax and remaining sections of the abdomen were used in the molecular analyses (nine male *Lu. longipalpis*).

**Fig. 1:**
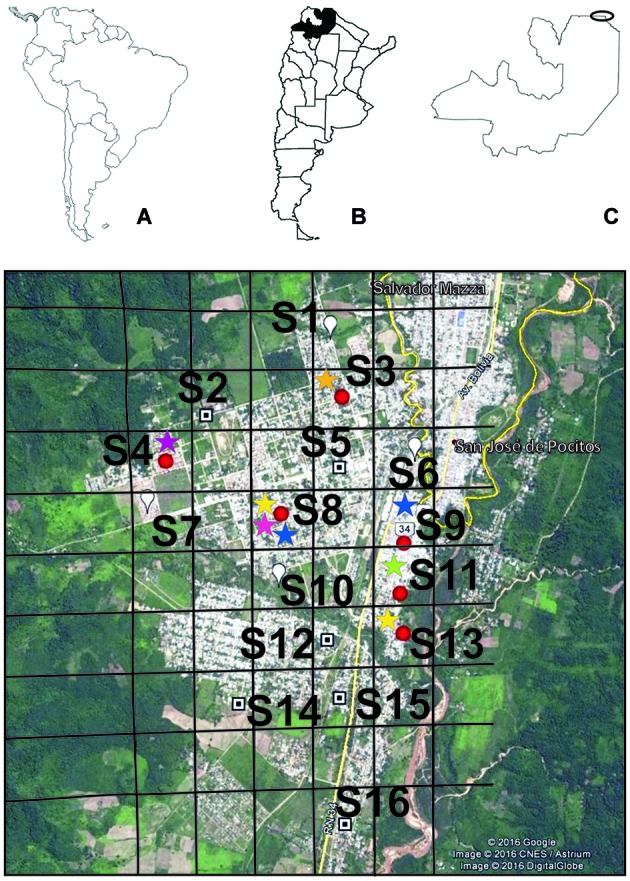
(A) South America. (B) Argentina, Salta province in black. (C) Salvador Mazza study area in Salta province. (D) Sampling sites (S1-S16) of *Phlebotominae* in Salvador Mazza. The red points are the sites with presence of *Lutzomyia longipalpis*. The colours of the stars represent the following haplotype codes: green, H43; purple, H44; yellow, H45; blue, H46; fuchsia, H57; orange, H58. Date of map © 2017 Google, Image © 2017 DigitalGlobe.

DNA was extracted using a DNA PuriPrep-S kit (Inbio Highway, Argentina) according to the manufacturer’s instructions. Cyt *b* fragment polymerase chain reaction (PCR) was performed as described previously (seven, for protocol references). DNA sequences were aligned using the MEGA version 7 software. Intrapopulation genetic diversity was evaluated with the number of mutations (h), number of segregating sites (*S*), number of unique sites (*Su*), number of haplotypes (*Nh*), *Hd*, nucleotide diversity (p), and nucleotide polymorphism index (q) using DnaSP version 5.10 software. Unique *Lu. longipalpis* haplotypes generated from Salvador Mazza were included in the database of Pech-May et al.^7^ The relationships among haplotypes were evaluated by constructing a minimum haplotype network using the median-joining method implemented in Network version 4.6 software, assuming that epsilon is zero. To evaluate the association between sites with presence or absence of *Lu. longipalpis* and those with presence or absence of environmental variables, we performed bivariate tests (chi-squared test). In all cases, Cramér’s V coefficient (CVC) was used to measure the association or independence among the considered variables, which has values between 0 (weak association) to one (strong association). The abundance of *Lu. longipalpis* was correlated with the number of dogs and chickens and percentages of different covers present in the plot of the studied site, such as trees, shrubs, cement, soil, and grass. InfoStat version 2016 software was used. All statistical tests were considered significant at a p-value ≤ 0.05.

A total of 65 individuals were captured with a sampling effort of 48 traps/night. The captured species were *Evandromyia cortelezzii* (Bréthes) and *sallesi* (Galvão & Coutinho) complex (43%), *Lu. longipalpis* (29%), *Nyssomyia neivai* (Pinto) (22%), and *Migonemyia migonei* (França) (6%). *Lu. longipalpis* was captured in 6/16 sampling sites (Fig. 1). The presence of *Lu. longipalpis* was significantly associated with the presence of the following variables: dogs (χ² = 15; p = 0.0001; CVC = 0.89), fruit trees (χ² = 6; p = 0.01; CVC = 0.58), organic detritus (χ² = 6; p = 0.01; CVC = 0.58), and chickens (χ² = 15; p = 0.0001; CVC = 0.89). The presence of ‘tree cover’ was the only significant variable associated with the abundance of the main vector of *L. infantum*, with at least 25% coverage (r = 0.53; p = 0.03). The most abundant site was S8 in the urban landscape.

Nine cyt *b* sequences of *Lu. longipalpis* revealed six haplotypes from Salvador Mazza (H43-H46 reported by Pech-May et al.^7^; H57 and H58 accession numbers: Mn228564 and Mn228565, respectively). The cyt *b* fragment length was 261 bp with 11 polymorphic sites and 250 conserved sites. The *Hd* was high (0.889 ± 0.091), while the nucleotide diversity (p = 0.013 ± 0.004) and nucleotide polymorphism index (*Ө* = 0.015 ± 0.002) were low. At site S8, three haplotypes were identified of four analysed specimens: two haplotypes shared with sites S9 and S13, while the other sites had unique haplotypes (Fig. 1). Regarding the haplotype network described previously,^7^ *Lu. longipalpis* from Salvador Mazza and those from the cities with higher *Hd* including Tartagal shared the H43, H44, H45, and H46 haplotypes, belonging to the Ar1 and Ar-Bra haplogroups (Fig. 2). The H46 haplotype between Tartagal and Salvador Mazza is at least two mutational steps from H18 of Bra haplogroup (Brazil). In contrast, the H57 haplotype of Salvador Mazza is two mutational steps from H39, the most frequent haplotype in Argentina, whereas those associated with the current dispersion from Brazil and Paraguay belong to the Ar2 haplogroup. Finally, H58 is one mutational step from H46 (Ar1 haplogroup) and two mutational steps from H5 and H39 (Ar-Bra and Ar2 haplogroups, respectively) (Fig. 2).

**Fig. 2:**
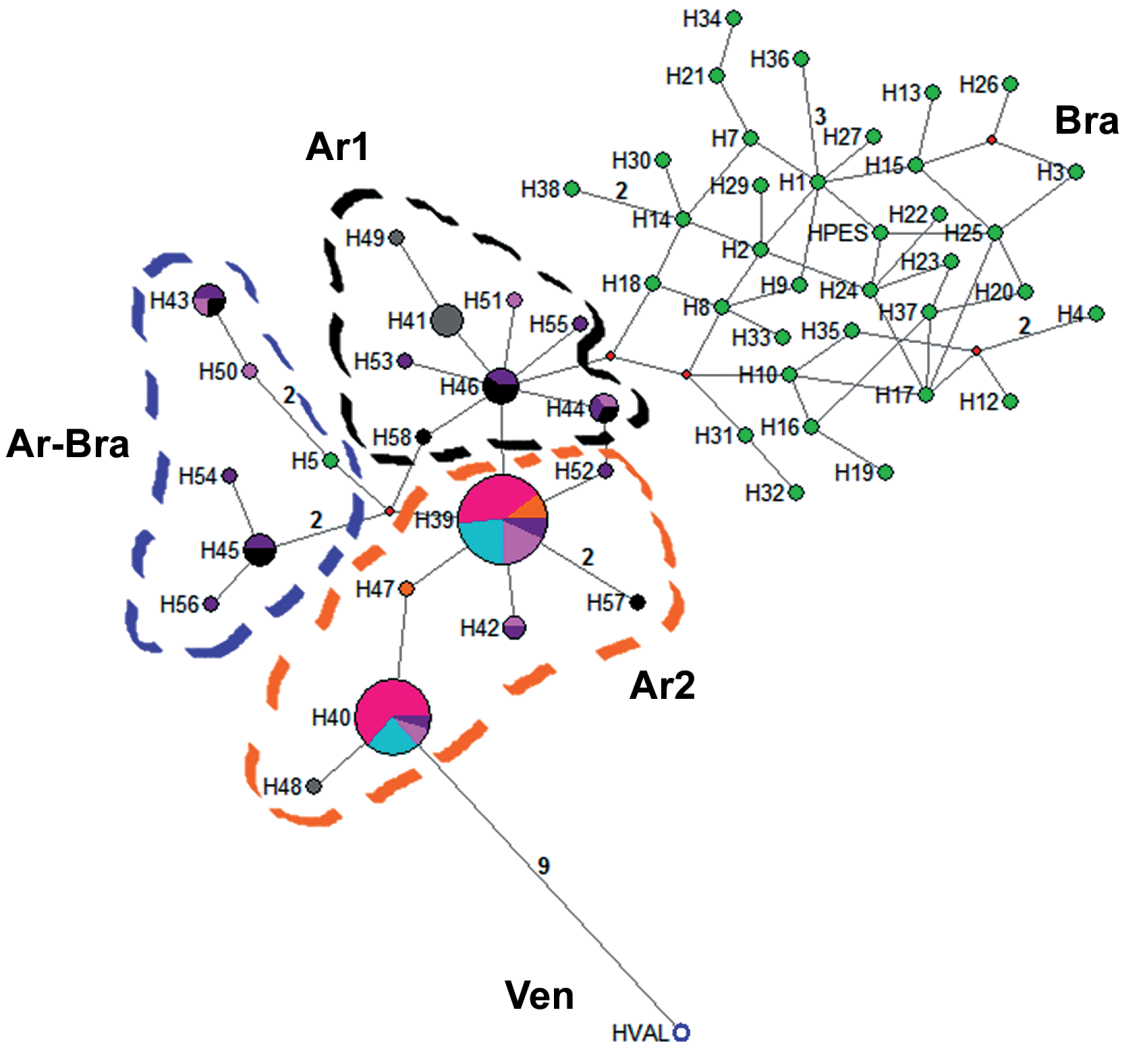
median-joining haplotype network of the *Lutzomyia longipalpis* complex based on 261 nucleotides of the 3’ region of the cyt *b* gene, obtained and modified by Pech-May et al*.* ^7^ The size of the circle corresponds to the frequency of each haplotype. Intermediary haplotypes (missing intermediate haplotypes) are shown as red circles. Each line connecting the haplotypes represents one mutational step, whereas the numbers along the lines are the total number of mutational steps between haplotypes. Colours indicate the sites: black (Salvador Mazza); orange (Clorinda); fuchsia (Corrientes); turquoise (Puerto Iguazú); grey (San Ignacio); pink (Santo Tomé); purple (Tartagal); white with blue outline (Venezuela); green (Brazil).

First, we initially report the presence of *Lu. longipalpis* in the northwestern Argentina-Bolivia border, in urban peridomestic environments in Salvador Mazza. The area had no records of this species in previous intensive sampling procedures,^4^ ^,^ ^5^ ^,^ ^10^ until reports in 2013 in Tartagal, 55 km south from the Salvador Mazza-Bolivia border.^6^ Second, the abundance of *Lu. longipalpis* is correlated with domestic blood sources, and heterogeneity of the urban landscape is associated with new urban foci.^11^ However, the low abundance of *Lu. longipalpis* in Salvador Mazza and Tartagal is suggestive of rural VL foci more than the urban foci of the recent urban dispersion wave, while the relative abundance of other species resembles the population assemblages of the rural xerophilic Chaco region, where the historical VL cases were reported in the early twentieth century.^12^ *Lu. longipalpis* of the northwestern Argentina-Bolivia border has a high *Hd* using cyt *b* gene, some haplotypes are found a few mutational steps from the other populations analysed from Argentina including even the recent emergent ones in the northcentral and northeast foci with low *Hd*, other haplotypes are shared with ‘ancient’ populations with higher *Hd*, and others are of their own but just a few mutational steps from the previous ones. The specimens of Salvador Mazza in the three haplogroups are already described in Argentina using two molecular markers (cyt *b* and ND4), the Ar1 and Ar2 haplogroups, exclusive to the country but also the Ar-Bra haplogroup, the last two being more closely related to the recent urban expansion of this species.^7^

Despite the fact that only 19 *Lu. longipalpis* were captured and the haplotypes of only nine male species were analysed, our results and those of previous studies obtained in the same area^6^ ^,^ ^7^ ^,^ ^13^ present the following issues: (a) Given the presence of *Lu. longipalpis* in Salvador Mazza, the risk of VL should be restated in the Bolivian Department of Tarija on the border with Argentina as the southernmost report of this species in Bolivia was in the caves of Torotoro, > 1000 km north from Salvador Mazza.^14^ (b) *Lu. longipalpis* in the Argentina-Bolivia border has a pattern of vector abundance, population assemblage, and associated report of scattered VL cases as in old rural xerophilic Chaco foci, but the urban vector distribution is typical of populations belonging to the recent expansions through humid forested areas. The absence of reported rural VL cases in northwestern Argentina for 80 years may be due to underreporting and the new awareness of the health system after the northeastern urban outbreak alert since 2006. (c) The high *Hd* and low nucleotide diversity suggest bottlenecks, followed by rapid population growth, while excessive low-frequency haplotypes are characteristic of relatively recent population expansion or selective sweep/hitchhiking. Similar values were observed in the long established populations in Argentina and Brazil.^7^ ^,^ ^13^ ^,^ ^15^

The contradictions observed in northwestern Argentina from *Lu. longipalpis* populations (Tartagal and Salvador Mazza) contribute to the establishment of the hypothesis of a recent urban expansion of *Lu. longipalpis* populations from Brazil-Paraguay that hybridize local populations during their expansion if there are no reproductive barriers. Hence, these populations have a rural species assemblage and epidemic pattern but are associated with urban environmental variables and distribution. These northwestern *Lu. longipalpis* populations have high *Hd* and low haplotype frequency, which are clustered with exclusive haplogroups of the country and the haplogroup shared with Brazil (Ar-Bra). Thus, if the crossbreeding hypothesis is true, the new populations could be exposed to adaptive pressure that selects not only the characteristics best fitted to the local environment already present in earlier populations but also new characteristics, such as the urbanisation capability from the populations in active dispersion. Regarding the possible path of expansion, there are informal comments regarding *Lu. longipalpis* in the xerophilic Chaco region of Paraguay and in southern Bolivia. The geographical gap of *Lu. longipalpis* records in the Chaco region from Brazil-Paraguay-Argentina originating in the eastern expansion could be related to the scarcity of records and low abundance in natural environments or actual unknown colonisation. The latter option could be from the Amazonian border of Bolivia-Paraguay-Brazil to the south, where the main vector of *L. infantum* seems to be *Lutzomyia cruzi* (Mangabeira), with the presence of *Lu. longipalpis*, and introgression and possible interhybridisation between these closer sibling species.^16^ de Oliveira et al.^17^ reported that the point of occurrence of *Lu. cruzi* in Bolivia is in the municipality of ‘El Carmen, Santa Cruz’, and the results of the modelling of potential ecological niche show a suitable area of the geographical region of ‘Gran Chaco’, in which Salvador Mazza is located.

This hypothesis of mixed ancestral rural, and new urban adapted and dispersive *Lu. longipalpis* populations has not only phylogeographic implications but also eco-epidemiological and control consequences, as the hybridised populations could be better adapted to local environments. The bionomy, vector capacity, and even insecticide susceptibility of these double-source populations could be different from those of the original population.
